# Do we have a lost generation of junior doctors: Effects of the COVID-19 pandemic on junior doctors’ resilience status, medical knowledge and medical skills

**DOI:** 10.1186/s12909-025-06819-2

**Published:** 2025-02-17

**Authors:** Nicola Katharina Kolb, Stephanie Keil, Johanna Huber

**Affiliations:** 1 Institut für Didaktik und Ausbildungsforschung in der Medizin, LMU Klinikum, Ludwig- Maximilians-Universität München, Pettenkofer Str. 8a, München, 80336 Bayern Deutschland; 2 Fakultät für Medizin, Universität Regensburg, Franz-Josef-Strauß-Allee 11, Regensburg, 93053 Bayern Deutschland

**Keywords:** Resilience, Competences, Junior doctors, COVID-19

## Abstract

**Background:**

At the onset of the COVID-19 pandemic, strict measures suspended face-to-face teaching at German universities, posing significant challenges for medical education. Practical, patient-centered training couldn’t be fully replaced by online formats, leading to skill deficits and increased stress among students. To date, no study has examined the impact of COVID-19 on the resilience and the competence of medical graduates so far. This study aims to address this knowledge gap by investigating the pandemic impact on resilience, medical competence, communication skills, and research competence of medical graduates.

**Methods:**

The study employed data from the “Bavarian Graduate Study of Medicine” (MediBAS), a cross-sectional evaluation survey conducted in cooperation with Bavarian universities and the Bavarian Institute for higher education research and planning. It targeted medical, dental, and veterinary graduates. The data were collected in two waves (2018/19 and 2022/23), with 1.114 human medicine graduates participating. The questionnaire assessed among others resilience, medical expertise, communication skills, and research competence. Statistical analysis involved descriptive statistics, correlation analysis, and Mann-Whitney-U tests due to non-normal data distribution.

**Results:**

The study analyzed self-assessed resilience, medical expertise, communication, and research skills of medical graduates from two waves. The findings demonstrated through descriptive statistics a decline in all competencies except research skills, which exhibited an increase. Correlation analysis revealed significant relationships between variables. Mann-Whitney-U tests revealed no significant differences between the waves in resilience (*p* =.079, *r* =.06), medical expertise (*p* =.117, *r* =.05), communication skills (*p* =.053, *r* =.07), or research competence (*p* =.106, *r* =.05).

**Conclusion:**

The study examined the impact of COVID-19 on medical graduates’ resilience, medical expertise, communication skills, and research competence. While there was a slight decline in resilience, medical expertise, and communication skills between the waves, there was an improvement in research competence. None of these changes were statistically significant. The findings suggest that the pandemic may have contributed to these trends by limiting practical experiences. No major negative impacts were found, suggesting no “lost generation” of doctors. The long-term effects of the changes remain uncertain due to the cross-sectional design and require further research.

## Background

At the onset of the COVID-19 pandemic in 2020, comprehensive measures such as stringent contact restrictions, hygiene protocols, and quarantine procedures were introduced to protect the health of the population [1]. Consequently, face-to-face teaching at universities and universities of applied sciences in Germany was prohibited, so that all classes and exams were promptly converted to pure online formats. This transition, however, presented significant challenges, particularly for medical training [2–5].

Unlike other fields, medical education consists largely of hands-on, patient-centered approaches such as laboratory exercises, bedside teachings, skills trainings, and the practical year (PJ) which require face-to-face teaching [2]. It was evident that hands-on, patient-centered teaching is indispensable to medical education [2]. Therefore, limited exemptions were made for hands-on and patient-oriented medical education and guidelines were defined (e.g., strict hygiene requirements, regular COVID-19 testing, restricted access to high-risk patients) to balance medical teaching requirements and the infection risks for patients involved in teaching situations. However, there was a decline in hands-on and patient-oriented teaching altered physician-patient interactions due to the stringent requirements of the clinical environment [6–9]. Students frequently voiced concerns regarding the paucity or substantial diminution of hands-on teaching, particularly bedside teaching, communication skills training, and practical clinical skills development [8].

Moreover, the PJ has undergone significant changes due to the pandemic, yet there is a paucity of literature addressing the extent of these modifications. Gisi et al. demonstrated that the first PJ was perceived by students as more stressful under the conditions of the pandemic than before the pandemic [10]. This perception was particularly pronounced regarding cancellations of PJ-accompanying teaching related to the pandemic, as well as the reduced patient contact during the PJ [10]. While students in the PJ, particularly those in the inaugural cohort under the influence of COVID-19, reported elevated levels of stress, they exhibited a higher degree of resilience and superior coping mechanisms when compared to their predecessors in the pre-pandemic era. This finding is consistent with other studies that have examined medical education under the conditions of COVID-19 outside the PJ [11, 12]. Collectively, these elements can be regarded as detrimental stressors, exerting a deleterious influence on the mental well-being of medical students.

Even before the pandemic, medical students consistently reported higher levels of stress, and a greater prevalence of related illnesses than students in other programs [13]. This phenomenon persists into the physicians’ professional lives, as they experience a higher prevalence of stress-related illnesses and a heightened risk of suicide when compared to the general population [14]. Research has identified enhancing mental resilience as a strategy to mitigate stress-related illnesses [15–17]. Resilience, defined as the capacity to cope with challenging circumstances or disaster without enduring permanent mental illness, is a crucial factor in this regard [18]. A multitude of exogenous and personal factors have been identified as contributors to enhanced resilience, including social support, self-efficacy expectations, optimism, and personal achievement [16]. Consequently, enhancing resilience and the factors that influence it can be regarded as a preventative measure to avert stress-related illnesses. To date, however, there has been a paucity of studies on the stability of resilience and the factors necessary for it in medical students [14, 19].

The Bavarian Graduate Study in Medicine (MediBAS) assessed resilience among Bavarian medical graduates in 2018 using the Connor-Davidson Resilience Scale, German version (CD-RISC 10), considering factors like gender, stress level, and certain professional skills. The findings revealed an average resilience score of 3.4, suggesting a high level of resilience among Bavarian medical graduates which is consistent with other studies of medical students in Germany [10, 20, 21]. A direct comparison of the scores is precluded by the utilization of disparate scales to measure resilience. Furthermore, the study by Kiesewetter et al. demonstrates a significant and positive correlation between the resilience of Bavarian medical graduates and their medical expertise and scientific competence [20]. Consequently, professional and scientific expertise appears to contribute positively to the development of resilience in medical graduates. Therefore, both constructs - competence development and resilience– ought to be considered in tandem. This interplay aligns with the theoretical model of professional identity formation (PIF), which describes the necessary skills, values, attitudes and socialization processes for the development of a good doctor [22].

The results of the studies indicate that medical students in Germany have well-developed resilience, i.e., they can cope well with difficult and crisis situations with the help of this resilience. However, a substantial body of research has revealed that physicians frequently report elevated levels of stress in their daily work, which is more prevalent compared to other professionals or the general population. Moreover, surveys have revealed that medical students have expressed concerns regarding the diminution of opportunities to practice their practical skills and abilities due to the COVID-19 pandemic.

The aim of this study is to contribute to closing the research gap by exploring the hypothesis that the satisfactory resilience measured by the MediBAS is sufficient to cope with the challenges posed by the pandemic. Additionally, it is investigated whether the pandemic influenced the medical competences learned at university. This leads to the following research questions: To what extent have the challenges posed by the COVID-19 pandemic, particularly during the PJ of medical studies, affected the resilience (RQ1), the medical expertise (RQ2), communication skills (RQ3), and research competence (RQ4) of graduates in their first year of practice? The study will explore the nature of these changes, their implications for medical education and training, and the direction in which these competencies have evolved.

## Methods

### Setting and study design

The present study is based on data from the “Bavarian graduate study of medicine”, also referred to as the “Bayerische Absolventenstudie Medizin” (MediBAS). This cross-sectional evaluation study was meticulously planned and conducted by the Bavarian State Institute for Higher Education Research and Planning (“Bayerisches Staatsinstitut für Hochschulforschung und Hochschulplanung”– short IHF). The IHF collaborated with the five Bavarian medical faculties offering degree programs in human and dentistry medicine, as well as one veterinary faculty. All faculties are organized in the Bavarian Network for Medical Education (Kompetenznetz Medizinlehre Bayern, short KMB) and belong to five Bavarian universities which are the Friedrich-Alexander-Universität Erlangen-Nürnberg (FAU), the Ludwig-Maximilians-Universität München (LMU), the Technische Universität München (TUM), the Universität Regensburg (UA), and the Julius-Maximilians-Universität Würzburg (JMU). In collaboration with the IHF, the faculties developed the survey instrument and disseminated it to all medical, dental and veterinary graduate students. The MediBAS was initiated during the fall/winter of 2015/16 and was established with a 3-year survey cycle. Consequently, the 2nd survey was conducted during the fall/winter 2018/19 period. The 3rd survey wave was postponed by one year due to COVID-19 and was conducted in the fall/winter of 2022/23. The target population for the survey consists of graduates in the fields of medicine, dentistry and veterinary medicine from the five Bavarian universities who have passed their 3rd state examination approximately six months to one and a half years prior to the survey. The participating faculties will contact the graduates via postal letter, providing information regarding the survey, including data protection, and a personal link to access the online survey. Participation in the study is voluntary and the compliance with the General Data Protection Regulation (GDPR) is guaranteed to graduates. Following the initial mailing, graduates who have not yet participated in the survey will receive a reminder letter. A second reminder with a paper questionnaire will be sent to the graduates’ home address at Christmas time. Upon completion of the survey, participants will be entered into a prize draw if they have made a commitment to participate, e.g. in the form of book vouchers. For a more thorough exposition of the study’s methodology, interested parties are encouraged to refer to the IHF’s report on the subject. The ethics committee of the Ludwig-Maximilians-Universität Munich has certified that the study is ethically unobjectionable (22–0665).

### Sample

The sample of this study consisted of two distinct waves from the MediBAS study, namely the 2nd wave and the 3rd wave. The study exclusively encompasses data from human medicine graduates. For the 2nd wave, 1610 human medical graduates were contacted, while for the 3rd wave 1218 graduates from human medicine were invited to participate in the evaluation study. Due to the postponement of the 3rd wave, participants in the 3rd wave experienced COVID-19 during their PJ at university. The 2nd wave included 613 medical graduates (375 female (61.2%), 195 male (31.8%), 43 no answer (7%)) who completed their studies between the 1st of October 2016 and the 30th of September 2017. The 3rd wave included 501 medical graduates (277 female (55.3%), 153 male (30.5%), 1 diverse (0.3%), 70 no answer (14.0%)) who completed their studies between 1st of April 2021 and 30th of March 2022.

### Questionnaire

The MediBAS questionnaire encompasses the following domains: (1) questions on the study program and assessment of competencies: medical expertise, communication and scientific competences (2) questions on the doctoral degree (3) questions about the career entry: employment, medical competence, specialist medical training and the collaboration between doctors and patients (4) personal information: resilience, personality & demographic data. Further information on the construction of the questionnaire is available in the “Feldbericht” (field report) of the IHF [23].

### Measurements

In order to test our hypotheses, we employed the following four measurement instruments which were included in both waves of the study.

#### Resilience

The measurement of resilience was conducted using the 10-Item Connor-Davidson Resilience Scale (CD-RISC), which was underwent translation into German [24]. An illustrative item is “Ich bin fähig mich anzupassen, wenn sich etwas verändert“ (I am able to adapt to change). Participants were instructed to respond to each item using a 5-point Likert scale, ranging from 1 “not true at all” to 5 “almost always true” The items were then aggregated to calculat a mean score, as the internal consistency of the scale was satisfactory, with coefficients ranging from.875 in the 2nd wave to.819 in 3rd wave.

#### Medical expertise

The medical expertise was assessed using the Freiburger Kompetenzfragebogen (Freiburger competence questionnaire) which contains 13 items [25]. An illustrative item is “Allgemeine Kenntnisse, Fähigkeiten und Fertigkeiten in der grundlegenden apparativen Diagnostik“ (General knowledge, skills and abilities in basic instrumental diagnostics). The evaluation of the items was conducted using a 5-point Likert scale from 1 “not at all” to 5 “to a very high degree”. Prior to calculating the mean score from 13 items, the reliability was assessed, yielding good reliability coefficients ranging from.887 (2nd wave) to.882 (3rd wave).

#### Communication skills

The communication skills scale was developed by medical communication experts of the medical faculty of LMU. It was constructed based on the “Communication Skills Attitude Scale (CSAS)” and includes 9 items [26]. An example item is “Fähigkeit, mit Patient*innen angemessen und sachlich zu kommunizieren” (Ability to communicate appropriately and objectively with patients). The mean score was calculated by combining all items, as the reliability was excellent, ranging from 0.912 (2nd wave) to 0.922 (3rd wave).

#### Research competence

The assessment of research competence was facilitated by “Munich Research Competence Scale”, a tool developed with the express purpose of evaluating medical research competence [27]. The scale consists of 10 items, including “Fähigkeit, das Ergebnis einer statistischen Hypothesenprüfung zu interpretieren” (Ability to interpret the result of a statistical hypothesis test). Prior to calculating the mean score of all items, the reliability was examined, resulting in an excellent Cronbach’s alpha of 0.930 for both waves. Further information on the used variables is available in the “Feldbericht” (field report) of the IHF [23].

### Statistical analysis

Prior to the analysis, the data set was prepared by removing participants who had ceased responding to the questionnaire on the first page. Those who had terminated their participation at a subsequent time point were retained in the data set. Subsequently, the statistical analysis was conducted using IBM SPSS Statistics, Version 29. Prior to hypothesis testing, a comprehensive analysis of descriptive statistics was conducted for each of the scales, namely resilience, communication skills, research competence, and medical expertise. This analysis encompassed the calculation of mean, standard deviation, and the number of correct cases. The analysis included only cases where all items on a scale were completed. The normality of each scale’s distribution was subsequently assessed using the Shapiro-Wilk test, with a *p*-value of less than 0.05 indicating non-normality. Furthermore, the Spearman-correlation coefficient was calculated between the scales within each wave to ascertain their inter-scale relationship. As the scales did not follow a normal distribution, non-parametric tests, such as the Mann-Whitney-U test, were employed.

To assess the hypotheses concerning potential differences between the 2nd and the 3rd wave within the context of resilience, medical expertise, communication skills, and research competence, the Man-Whitney-U test was employed. The alpha level was set at 5%. To ascertain the impact of group disparities, the effect size r was computed for non-normally distributed data.

## Results

### Descriptive statistics

Tables 1 and 2 present the descriptive statistics of all scales for the 2nd and the 3rd wave, respectively. It should be noted that not all participants answered all questions, which is reflected in the varying number of observations (N) reported in the tables. The findings indicated an enhancement in the research competence of medical graduates from the 2nd wave to the 3rd wave, accompanied by a decline in resilience, communication skills, and medical expertise. The Shapiro-Wilk test was employed to assess the normality distribution of the scales. The results indicated that only medical expertise in the 2nd wave exhibited a normal distribution (*p* =.114), while the remaining scales did not conform to a normal distribution. Furthermore, the inter-scale correlations were examined within each respective wave. The analysis revealed that all correlations were significant in both waves. In the 2nd wave, resilience exhibited a medium correlation with medical expertise and a low correlation with all other scales. Additionally, medical expertise demonstrated a high correlation with communication skills and a high correlation with research competence. In the 3rd wave, resilience exhibited a low (communication skills and research competence) to medium correlation (medical expertise) with the other scales. The scales medical expertise and communication skills exhibited a high correlation with each other.

**Table 1:** Descriptive statistics and bivariate correlations of main scales - Wave 2

Variable	*N*	Missing	Range	M	SD	Shapiro-Wilk	1	2	3	4
1. Resilience	409	204	1.5-5	3.73	0.628	< 0.001	-			
2. Medical Expertise	578	35	1.62-5	3.36	0.598	0.133	0.324*	-		
3. Communication Skills	430	183	1–5	2.85	0.825	0.006	0.266*	0.591*	-	
4. Research Competence	571	42	1–5	2.73	0.802	0.002	0.233*	0.533*	0.534*	-

Note. *significant *p* <.001

**Table 2:** Descriptive statistics and bivariate correlations of main variables - Wave 3

Variable	*N*	Missing	Range	M	SD	Shapiro-Wilk	1	2	3	4
1. Resilience	420	81	1.7-5	3.67	0.504	0.002	-			
2. Medical Expertise	457	44	1-4.92	3.30	0.608	0.041	0.303*	-		
3. Communication Skills	403	98	1–5	2.75	0.878	< 0.001	0.185*	0.600*	-	
4. Research Competence	435	66	1–5	2.82	0.837	0.013	0.249*	0.497*	0.410*	-

Note. *significant *p* <.001

### Differences between the two waves

The objective of this investigation was to ascertain whether there is a discrepancy in self-assessed resilience, medical expertise, communication skills, and research competence between medical graduates from the 2nd and 3rd survey waves. To assess these variables, a Mann-Whitney-U-test was employed, given that the Shapiro-Wilk test revealed the data did not follow a normal distribution (see Tables 1 and 2).

**Fig. 1:**
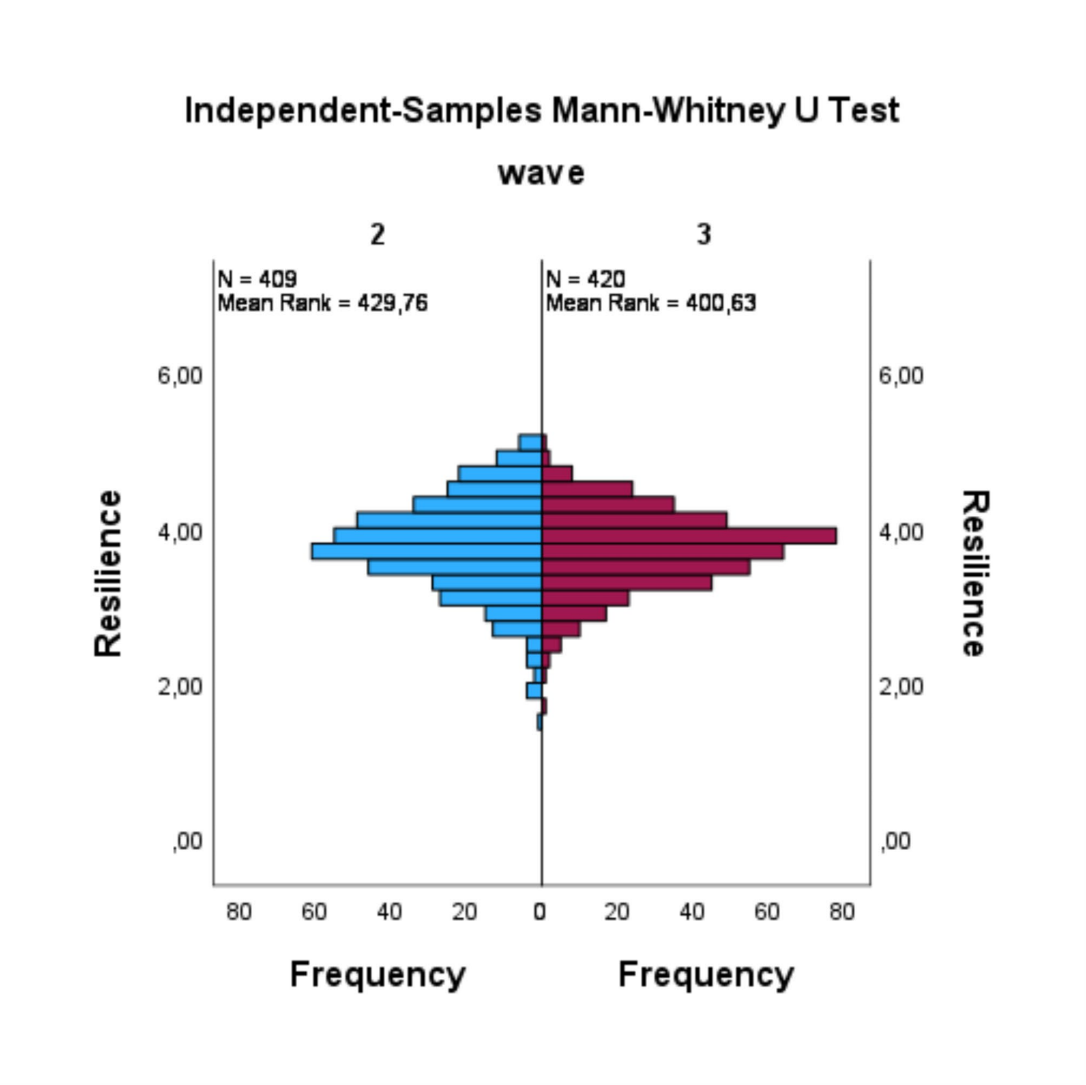
Mann-Whitney-U test for resilience

**Fig. 2:**
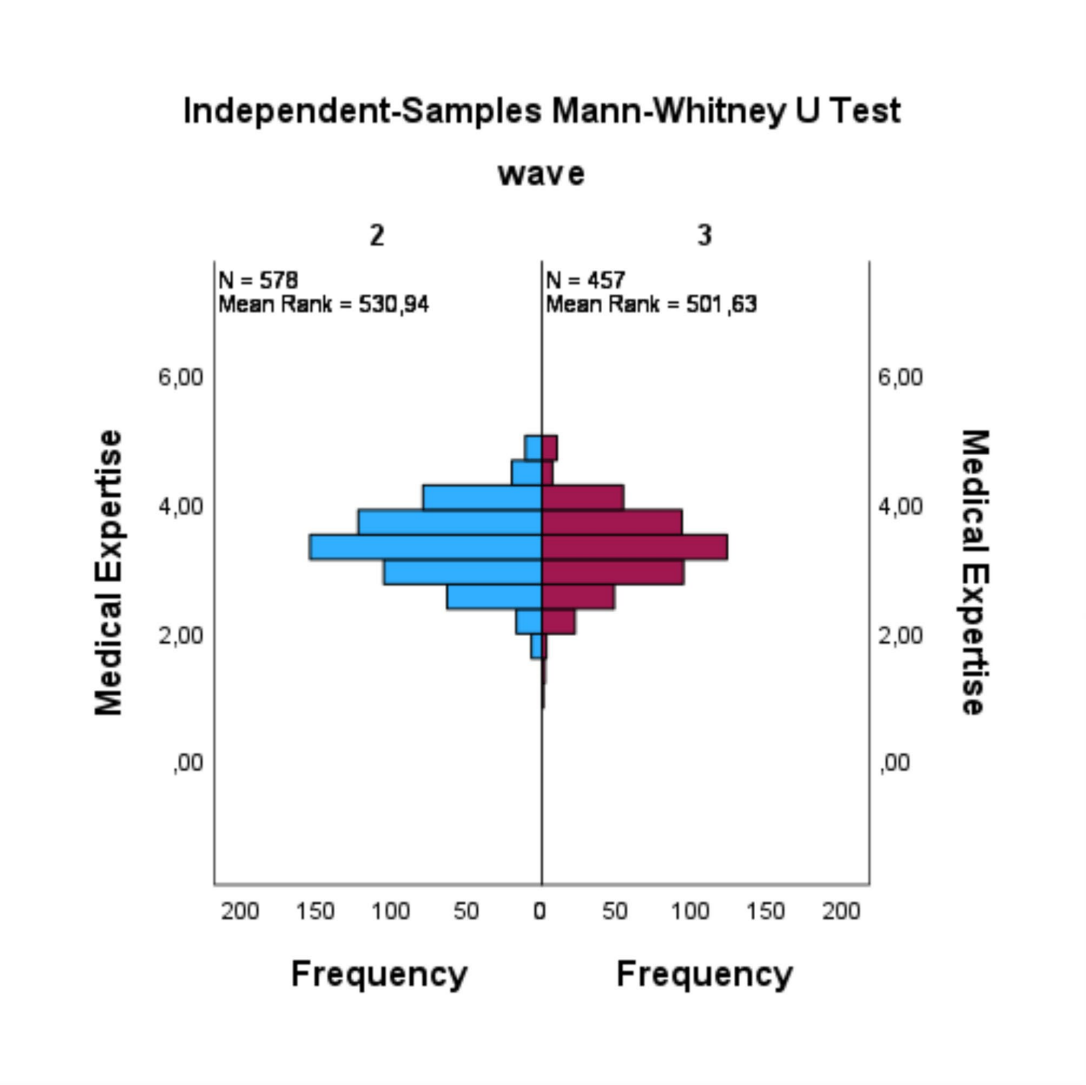
Mann-Whitney-Utest for medical expertise

**Fig. 3:**
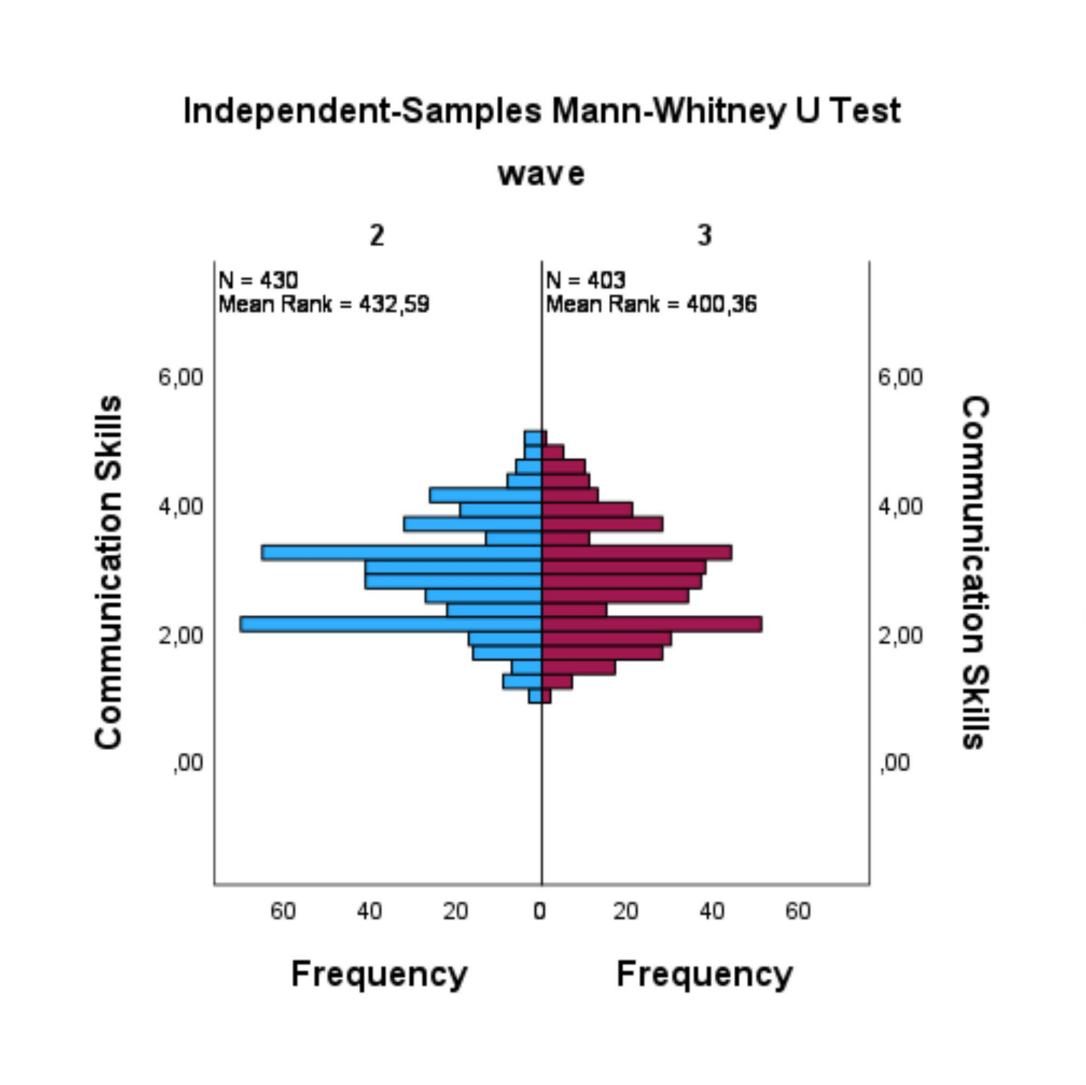
Mann-Whitney-U test for communication skills

**Fig. 4:**
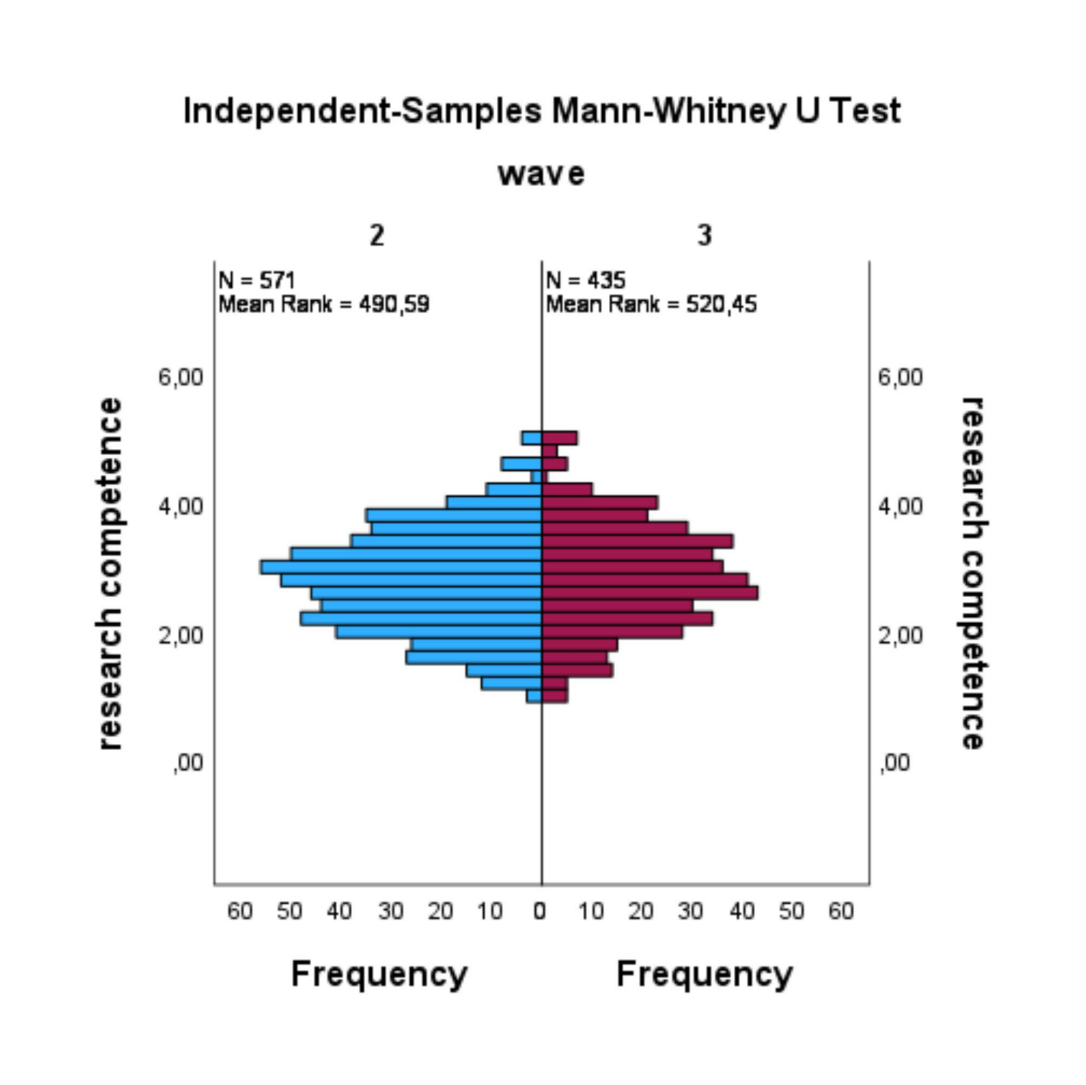
Mann-Whitney-Utest for research competence

The findings concerning resilience (RQ1) indicated an absence of a statistically significant difference between the two waves (U = 79854.5, *p* =.079, *r* =.06). The distribution revealed that the mean rank of resilience in the 2nd wave was higher than that in the 3rd wave, with more elevated values and more depressed values (see Fig. 1). In the context of medical expertise (RQ2), the results indicated that there was no significant difference between the two waves (U = 124591.5, *p* =.117, *r* =.05). In this case, the distribution demonstrated that medical expertise in the 2nd wave had a higher mean rank compared to the 3rd wave (see Fig. 2). Additionally, there were no statistically significant differences observed between the 2nd wave and the 3rd wave in terms of communication skills (RQ3) (U = 79940.5, *p* =.053, *r* =.07) and research competence (RQ4) (U = 131564, *p* =.106, *r* =.05). The distribution of both waves for communication skills exhibited a decrease in the mean rank from the 2nd to the 3rd wave, while both waves demonstrated some peaks that were more pronounced in the 2nd wave (see Fig. 3). In contrast, the distribution of research competence exhibited an increase from the 2nd wave to the 3rd wave in the mean rank (see Fig. 4).

## Discussion

The objective of this study was to examine the impact of the COVID-19 pandemic on the self-rated resilience, medical expertise, communication skills, and research competence of medical graduates.

The descriptive results indicate that only the graduates’ research competence demonstrated an increase from the pre-pandemic period (2nd wave) to pandemic period (3rd wave); however, this increase was not statistically significant. Furthermore, a decline in the self-rated resilience, medical expertise and communication skills of medical graduates was observed from the 2nd wave to the 3rd wave. The decline in communication skills is not statistically significant, but it does exhibit a small effect size.

A further analysis of the distribution of mean values on the resilience scale reveals that the values observed in the 2nd wave are more dispersed, with a notable presence of values in the lower range of the scale. A more pronounced skewed distribution of resilience values is evident in the 2nd wave compared to the 3rd wave, although the mean value for the 2nd wave exceeds that of the 3rd wave. In contrast, the 3rd wave exhibited a more concentrated distribution of resilience values around the scale mean, with a notable absence of values in the upper range of the scale compared to the 2nd wave. This observation may be indicative of two factors. Firstly, in 3rd wave still many resilient graduates took part in the survey overall and less resilient graduates did not feel addressed by the survey and therefore did not take part in the survey. Concurrently, the mean resilience score exhibited a decline in the 3rd wave compared to the 2nd wave, indicating a modest impact of the pandemic on medical graduates’ resilience, nearly reaching a level of statistical significance.

The underlying reasons for this phenomenon are not immediately apparent and may be multifaceted. The COVID-19 pandemic may have resulted in a subset of students, particularly those who were less resilient, failing to complete their degree within the standard period of study. Consequently, these students may not have been included in the present study. A comparative analysis of the data reveals that 38.27% of graduates completed their studies within the standard period of study in the 2nd wave which predates the advent of COVID-19. Conversely, in the survey conducted during the 3rd wave, which coincided with COVID-19, only 29.48% of graduates completed their studies within the standard period of study. This decline, amounting to almost 10% of the sample, is associated with a decrease in self-rated resilience values compared to the 2nd wave. Prolonged study periods can be perceived as a stress factor, as students may face questions regarding financing their studies, potentially leading to a decline in resilience. In addition to the extended study duration, the transition to an alternative teaching environment, characterized by its potential to induce greater stress compared to traditional teaching setting [28–30], may have contributed to the observed decline in resilience. The findings indicate that the impact of COVID-19 may have influenced the duration of graduate studies, with a potential decline in resilience observed from the 2nd wave to the 3rd wave. Given the limitations of our sample, it is plausible that a greater proportion of graduated had to extend their studies during the 3rd wave either due to ongoing studies at the time of survey administration or due to reluctance to participate in the survey.

Another potential explanation for the decline in resilience scores among 3rd wave graduates may be found in the analogous results observed on the medical expertise scale. This scale, akin to resilience, exhibited median to upper middle rankings in both survey waves. However, in the 3rd wave these values were absent. However, it is noteworthy that these values exhibited a decline from the 2nd wave to the 3rd wave, though this decline did not reach statistical significance. Nonetheless, the effect size of this decline was small. It can be hypothesized that the impact of COVID-19 and its repercussions on the PJ have contributed to the observed decline in medical expertise. A similar pattern is observed in the communication skills scale. In this case, too, the values underwent a decline from the 2nd to the 3rd wave, though this decline did not attain a statistically significant level with 0.05 with a small effect size. The overall values of the communication skills scale are situated within the lower middle to the upper middle range of the scale. One potential explanation for the decline in both scales could be the reduced opportunities for practicing and applying medical and communication skills during the PJ of medical training for graduates of the 3rd survey wave. For instance, there may been a lack of application of clinical skills or fewer opportunities to interact with patients, as final year students were assigned to care for patients with COVID-19. The scales in question inquire about specific competencies within the clinical milieu, such as communication of mistakes and knowledge in basic diagnostics. It is plausible that the graduates of the 3rd wave encountered limitation in fully applying these skills during their final year, which might have contributed to their self-perception of inferiority in these domains. This slight deterioration in medical and communication skills does appear to lead to a poorer resilience. A positive correlation has been observed between both domains of competence and resilience. This finding suggests that these competencies may act as either a protective factor for the development of resilience when having high values like in 2nd wave or a negative factor for the development of resilience when lower values are there like 3rd wave despite effective coping with a challenging situation such as the management of COVID-19 pandemic.

A similar pattern is evident in the research competence data. The scale’s mean values exhibited a slight improvement from the 2nd wave to the 3rd wave, though this change was not statistically significant. The overall distribution of values on the scale aligns with the findings observed in the communication scale, indicating a tendency towards the upper middle range of the scale. However, a closer examination reveals a slightly more even distribution in the 3rd wave. The absence of a statistically significant change in the values for research competence is likely attributable to the fact that the instruction in scientific competence remained relatively unchanged between the 2nd and the 3rd waves. The 3rd wave of graduates experienced the theoretical lessons regularly, without the restrictions imposed by the pandemic. The slight improvement in the values could be attributed to the direct application of scientific knowledge in the treatment of patients with COVID-19 during the PJ, under conditions of the pandemic. This experience enabled the graduates to develop a more profound understanding of the generation and application of scientific evidence. Furthermore, the students’ understanding of the significance of research has increased. Additionally, a positive and significant correlation between scientific competence and resilience, though the magnitude of this correlation is low. The presence of scientific competence has been demonstrated to exert a positive and significant influence on resilience, as evidenced by the heightened sense of confidence exhibited by young doctors in the implementation of evidence-based medicine. However, the correlation between the medical expertise and resilience appears to be more significant, with scientific expertise correlating strongly with medical expertise.

A comprehensive analysis reveals that the three competence levels (medical expertise, communication skills and research competence) exert a significant influence on resilience, thereby contributing to its enhancement. The extent to which a selection bias exists remains unresolved. On the one hand, two areas of competence exhibited a decline from the 2nd wave to the3rd wave; however, this decline was not statistically significant, suggesting that the sample does not predominantly consist of high performers. This is further evidenced by the observation that the 3rd wave includes nearly 10% more graduates who did not complete their studies within the standard period of study. Conversely, the domain of medical expertise is appraised very highly by the graduates from both survey waves. However, when taking into account all the factors, it cannot be disregarded that our sample may contain a significant proportion of highly motivated and high-performing graduates. Consequently, the prevalence of individuals with low resilience or those disproportionately impacted by the pandemic may be underestimated. Beyond the high motivation levels exhibited by the graduates, it remains uncertain whether these individuals possessed high values during their medical studies or if these values underwent changes due to the impact of the pandemic. The following limitations should be considered: The study was conducted exclusively at universities in Bavaria, excluding other institutions in Germany. The restriction to Bavarian universities precludes the possibility of generalizing the results to other universities in Germany. Additionally, all scales are measured on a self-assessment scale. Objective measurements, which are not influenced by personal desire, opinion, or social desire, may yield different results. Nonetheless, it is important to acknowledge the role that self-assessments of competences play in the development of self-efficacy. Therefore, they should be considered in conjunction with objective competence measurements [31, 32]. In the context of the utilized scale, an additional limitation is that the competence scales are recent inventories, particularly the research competence and communication competence, which necessitate further validation. Finally, the response rate for the survey is between 38% (2nd wave) and 41% (3rd wave), which is not high but is consistent with the rates observed in online surveys in the field of evaluation [33]. Notably, the response rate during the period of the pandemic exceeded the rates observed prior to the onset of the virus. This suggest that despite the stressful nature of the pandemic, graduates may have been motivated to participate in online surveys.

## Conclusions

Notwithstanding aforementioned limitations, the study demonstrated reciprocal dependencies between resilience and the various competences, in terms of medical expertise, communication skills, and research competence, which exhibit varying degrees of interconnection. Concurrently, these competences are influenced by COVID-19 in disparate ways. While resilience, medical expertise and communication skills exhibited a decline from 2nd wave to 3rd wave, research competence demonstrated an increase through the waves. Consequently, the competences and the resilience are influenced by COVID-19 and the corresponding challenges. As cross-sectional data is analyzed, it is not possible to determine causality and potential interactions between resilience and the examined competences. This would require a multivariate model. The study’s objective was to determine whether the COVID-19 pandemic has led to a loss of a generation of doctors. However, based on the findings of this study, it can be concluded that, at least in the present, this has not been the case. Nevertheless, the results of this study indicate the presence of certain adverse changes which were likely precipitated by the effects of COVID-19. However, the long-term implications of the changes remain uncertain and require further investigation through additional longitudinal studies.

As COVID-19 already had an effect on resilience and the competences, it is important to take these findings into consideration when planning the curriculum and adding new teaching formats which could affect the learning of competences or new training like a resilience training which could improve the resilience [21, 34]. At the same time, the broader concept of PIF should be given greater consideration in curriculum development with regard to strengthening resilience, so that resilience is not trained as an isolated competence.

Subsequent studies could investigate the extent to which the findings of this study can be generalized to graduates from other universities in Germany or other countries. A further question pertains to the integration of objective instruments for measuring competencies into the Bavarian Graduate Study. Additionally, there is the possibility of conducting longitudinal surveys of MediBAS participants to better evaluate the impact of the pandemic, as some respondents have provided their contact information for future surveys. However, this the sample size is limited and the challenge of panel mortality in longitudinal studies remains a concern.

## Data Availability

The MediBAS data can be requested from the Bavarian Institute of Higher Education Research and Planning (www.ihf.bayern.de).
